# Genome-wide colocalization of RNA–DNA interactions and fusion RNA pairs

**DOI:** 10.1073/pnas.1819788116

**Published:** 2019-02-04

**Authors:** Zhangming Yan, Norman Huang, Weixin Wu, Weizhong Chen, Yiqun Jiang, Jingyao Chen, Xuerui Huang, Xingzhao Wen, Jie Xu, Qiushi Jin, Kang Zhang, Zhen Chen, Shu Chien, Sheng Zhong

**Affiliations:** ^a^Department of Bioengineering, University of California, San Diego, La Jolla, CA 92093;; ^b^Institute of Engineering in Medicine, University of California, San Diego, La Jolla, CA 92093;; ^c^Division of Biological Sciences, University of California, San Diego, La Jolla, CA 92093;; ^d^Department of Biochemistry and Molecular Biology, College of Medicine, The Pennsylvania State University, Hershey, PA 17033;; ^e^Department of Ophthalmology, University of California, San Diego, La Jolla, CA 92093;; ^f^Department of Diabetes Complications and Metabolism, Beckman Research Institute, City of Hope, Duarte, CA 91010;; ^g^Department of Medicine, University of California, San Diego, La Jolla, CA 92093

**Keywords:** fusion transcripts, RNA–DNA interactions, RNA-poise model

## Abstract

Fusion transcripts are used as biomarkers in companion diagnoses. Although more than 15,000 fusion RNAs have been identified from diverse cancer types, few common features have been reported. Here, we compared 16,410 fusion transcripts detected in cancer (from a published cohort of 9,966 tumor samples of 33 cancer types) with genome-wide RNA–DNA interactions mapped in two normal, noncancerous cell types [using iMARGI, an enhanced version of the mapping of RNA–genome interactions (MARGI) assay]. Among the top 10 most significant RNA–DNA interactions in normal cells, 5 colocalized with the gene pairs that formed fusion RNAs in cancer. Furthermore, throughout the genome, the frequency of a gene pair to exhibit RNA–DNA interactions is positively correlated with the probability of this gene pair to present documented fusion transcripts in cancer. To test whether RNA–DNA interactions in normal cells are predictive of fusion RNAs, we analyzed these in a validation cohort of 96 lung cancer samples using RNA sequencing (RNA-seq). Thirty-seven of 42 fusion transcripts in the validation cohort were found to exhibit RNA–DNA interactions in normal cells. Finally, by combining RNA-seq, single-molecule RNA FISH, and DNA FISH, we detected a cancer sample with EML4-ALK fusion RNA without forming the EML4-ALK fusion gene. Collectively, these data suggest an RNA-poise model, where spatial proximity of RNA and DNA could poise for the creation of fusion transcripts.

## Significance

It remains formidable to predict what unreported RNA pairs can form new fusion transcripts. By systematic mapping of chromatin-associated RNAs and their respective genomic interaction loci, we obtained genome-wide RNA–DNA interaction maps from two noncancerous cell types. The gene pairs involved in RNA–DNA interactions in these normal cells exhibited strong overlap with those with cancer-derived fusion transcripts. These data suggest an RNA-poise model, where the spatial proximity of one gene’s transcripts and the other gene’s genomic sequence poises for the creation of fusion transcripts. We validated this model with 96 additional lung cancer samples. One of these additional samples exhibited fusion transcripts without a corresponding fusion gene, suggesting that genome recombination is not a required step of the RNA-poise model.

Fusion transcripts are associated with diverse cancer types and have been proposed as diagnostic biomarkers (1–3). Companion tests and targeted therapies have been developed to identify and treat fusion-gene defined cancer subtypes (2, 4). Efforts of detection of fusion transcripts have primarily relied on analyses of RNA sequencing (RNA-seq) data (1, 3, 5–8). A recent study analyzed 9,966 RNA-seq datasets across 33 cancer types from The Cancer Genome Atlas (TCGA) and identified more than 15,000 fusion transcripts (4).

Despite the large number of gene pairs in identified fusion transcripts, it remains formidable to predict what unreported pair of genes may form a new fusion transcript. Recent analyses could not identify any distinct feature of fusion RNA forming gene pairs (9). Here, we report a characteristic pattern of the 2D distribution of the genomic locations of the gene pairs involving RNA–DNA interactions that provides insights into understanding the creation of fusion transcripts.

Chromatin-associated RNAs (caRNAs) provide an additional layer of epigenomic information in parallel to DNA and histone modifications (10). The recently developed mapping of RNA–genome interactions (MARGI) technology enabled identification of diverse caRNAs and the respective genomic interacting locations of each caRNA (6). In this work, we developed an improved MARGI experimental pipeline called iMARGI. Compared with MARGI, iMARGI reduced the required amount of input cells by 100-fold to ∼5 million cells. We used iMARGI to map RNA–DNA interactions in human embryonic kidney (HEK) and human foreskin fibroblast (HFF) cells. The detected RNA–DNA interactions often appeared on the gene pairs involved in cancer-derived fusion transcripts. The widespread RNA–DNA interactions on the gene pairs involved in fusion transcripts suggest a model wherein the RNA of gene 1 by interacting with the genomic sequence of gene 2 is poised for being spliced into gene 2’s nascent transcript and thus creating a fusion transcript. Consistent with this model, we identified an RNA fusion in a new cancer sample that does not involve the creation of a fusion gene.

## Results

### Characteristics of Genome-Wide RNA–DNA Interaction Maps.

**Fig. 1.**
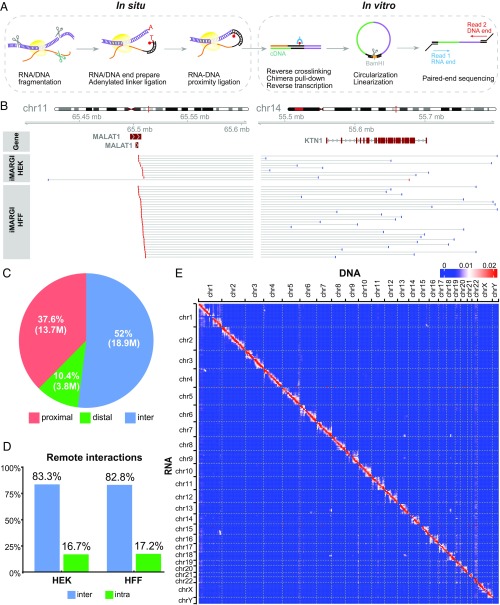
Overview of iMARGI method and data. (*A*) Schematic view of iMARGI experimental procedure. (*B*) An example of interchromosomal read pairs (horizonal lines), where the RNA ends (red bars) were mapped to the MALAT1 gene on chromosomal 11, and the DNA ends (blue bars) were mapped to chromosome 14 near the KTN1 gene (blue bars). (*C*) Proportions of proximal, distal, and interchromosomal read pairs in a collection of valid RNA–DNA interaction read pairs. M: million read pairs. (*D*) Ratios of inter- and intrachromosomal read pairs in HEK and HFF cells after removal of proximal read pairs. (*E*) Heatmap of an RNA–DNA interaction matrix in HEK cells. The numbers of iMARGI read pairs are plotted with respect to the mapped positions of the RNA end (row) and the DNA end (column) from small (blue) to large (red) scale, normalized in each row.

To systematically characterize caRNAs and their genomic interaction locations, we developed iMARGI, an enhanced version of the MARGI assay (10). The main difference between iMARGI and MARGI is that iMARGI carries out the ligation steps in situ (Fig. 1*A*), whereas MARGI performs these ligation steps on streptavidin beads. We applied iMARGI to HEK and HFF cells to yield 361.2 million and 355.2 million 2 × 100-bp paired-end sequencing read pairs, respectively. These resulted in 36.3 million (HEK) and 17.8 million (HFF) valid RNA–DNA interaction read pairs, in which ∼35%, 10%, and 55% were proximal, distal, and interchromosomal interactions, respectively (Fig. 1*C* and *SI Appendix*, Fig. S1*A*). The proximal and distal interactions were defined as the intrachromosomal interactions where the RNA end and DNA end were mapped to within and beyond 200 kb, respectively. Following Sridhar et al. (10), we removed proximal read pairs from further analysis because proximal interactions likely represent interactions between nascent transcripts and their neighboring genomic sequences. Hereafter, we refer to the union of distal and interchromosomal interactions as remote interactions. The rest of this paper deals only with remote interactions.

**Fig. 2.**
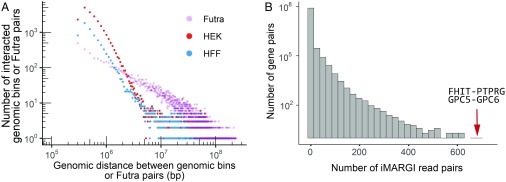
Summary of iMARGI data. (*A*) The number of genomic bin pairs with 10 or more iMARGI read pairs (*y* axis) is plotted against the genomic distance between the pair of genomic bins (*x* axis) in HEK cells (red circles) and HFF cells (blue circles). For comparison, the number of fusion transcript-contributing RNA (Futra) pairs (*y* axis) derived from 9,966 cancer samples is plotted against the genomic distance (*x* axis) between the genes involved in the fusion (purple circles). (*B*) Histogram of the number of iMARGI read pairs of every gene pair (*x* axis) across all of the intrachromosomal gene pairs with genomic distance >200 kb in HEK cells. Arrow: the gene pairs with the largest and the second largest number of iMARGI read pairs.

Among the remote RNA–DNA interactions, both cell types exhibited an ∼1:5 ratio of intra- and interchromosomal interactions (Fig. 1*D*). The 2D map of RNA–DNA interactions exhibited more interactions near the diagonal line (Fig. 1*E* and *SI Appendix*, Fig. S1*D*). Within each chromosome, the number of iMARGI read pairs negatively correlated with their genomic distances (Fig. 2*A*, red and blue circles).

### Comparison of iMARGI with MARGI.

We compared iMARGI with the MARGI technology previously described (10). iMARGI requires only ∼5 million cells to start the experiment, whereas MARGI requires ∼500 million cells. MARGI has two technical variations called pxMARGI and diMARGI, which differ by the degree of chromatin fragmentation (10). We compared the iMARGI, pxMARGI, and diMARGI datasets generated from HEK293T cells. These datasets had roughly comparable amounts of raw read pairs (*SI Appendix*, Table S1).

First, we compared the distribution of the read pairs. iMARGI and pxMARGI produced similar amounts of valid interchromosomal (∼19 million) and distal (1–4 million) read pairs (*SI Appendix*, Table S1). diMARGI generated many fewer valid interchromosomal (∼0.5 million) and distal (∼26,000) read pairs.

Second, we compared by the numbers of discovered caRNAs. Under the smallest possible threshold (1 valid read pair), iMARGI revealed a similar amount of caRNAs to that of pxMARGI, with mRNA, lincRNA, pseudogene RNA, and antisense RNA as the most abundant types of caRNAs (*SI Appendix*, Fig. S2). diMARGI revealed severalfold fewer caRNAs in every RNA type (*SI Appendix*, Fig. S2), consistent with its many fewer usable read pairs.

Third, we compared by the “RNA attachment level” (10) on every genomic segment. We segmented the genome into 100-kb bins and counted the number of read pairs with the DNA end mapped to each bin. iMARGI and pxMARGI exhibited a strong correlation (Pearson correlation = 0.88, *P* value <2.2×10^−16^; *SI Appendix*, Fig. S3*A*). The correlation increased with the bin size (*SI Appendix*, Fig. S3 *B* and *C*). diMARGI data exhibited much weaker correlation to iMARGI data (*SI Appendix*, Fig. S3 *D*–*F*), likely also due to its very small amount of usable read pairs.

### The Most Significant RNA–DNA Interactions Colocalized with the Gene Pairs Forming Fusion Transcripts in Cancer.

We set off to identify the most significant distal RNA–DNA interactions from the iMARGI data. Excluding extremely abundant noncoding RNAs, such as XIST, the top gene pair with the largest amount of interchromosomal and distal iMARGI read pairs in HEK cells was FHIT-PTPRG (Fig. 2*B* and *SI Appendix*, Fig. S4*A*). Investigating this gene pair, we found the reporting of FHIT-PTPRG fusion transcripts from kidney, liver, head and neck, lung, and prostate cancers (7). The second largest amount of interchromosomal and distal iMARGI read pairs was from GPC5-GPC6 (Fig. 2*B* and *SI Appendix*, Fig. S4*B*). Fusion transcripts from this second-ranked gene pair were reported from liver and prostate cancers (7). Notably, 5 of the top 10 gene pairs were reported as fusion transcripts in cancers (1, 7). These findings led us to systematically analyze the relationship between RNA–DNA interactions and fusion transcripts.

### Nonuniform Distribution of the RNA Pairs Contributing to Fusion Transcripts.

**Fig. 3.**
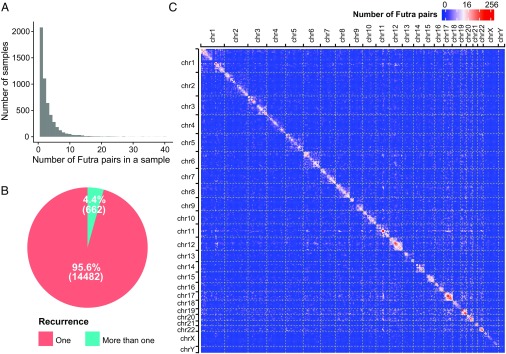
Overview of the 15,144 Futra pairs derived from 33 cancer types. (*A*) Distribution of the number of detected Futra pairs in each sample across the 9,966 cancer samples. (*B*) Pie chart of the numbers of Futra pairs that appeared in only one cancer sample (nonrecurring Futra pairs, red) and in multiple cancer samples (recurring Futra pairs, green). (*C*) The genomic distribution of Futra pairs. Genomic coordinates of all chromosomes are ordered on rows and columns. The numbers of Futra pairs of corresponding genomic positions are shown in a blue (small) to red (large) color scheme. Bin size: 10 Mb.

We asked whether there is any global characteristic of genome-wide distribution of the RNA pairs that contribute to fusion transcripts. To this end, we subjected the previously reported 16,410 fusion transcripts that were derived from 9,966 samples across 33 cancer types from TCGA project to further analysis (4). On average, there were two fusion transcripts per sample (Fig. 3*A*). The 16,410 fusion transcripts corresponded to 15,144 unique RNA pairs. Hereafter we refer to these gene pairs as fusion transcript-contributing RNA pairs (“Futra pairs”). More than 95% (14,482 of 15,144) of Futra pairs occurred only in 1 sample of the 9,966 samples analyzed (Fig. 3*B*). These data confirmed the scarcity of recurrent gene pairs in fusion transcripts (11, 12).

**Fig. 4.**
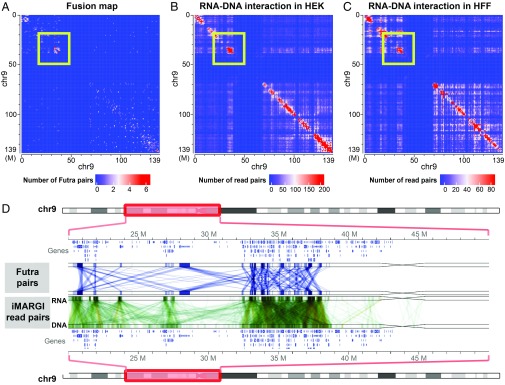
Comparison of Futra pairs and iMARGI read pairs. (*A*) Distribution of intrachromosomal Futra pairs on chromosome 9. Genomic coordinates are plotted from top to bottom (rows) and from left to right (columns). (*B* and *C*) RNA–DNA interaction matrices in HEK cells (*B*) and HFF cells (*C*). The numbers of iMARGI read pairs are plotted with respect to the mapped positions of the RNA end (rows) and the DNA end (columns) on chromosome 9 from small (blue) to large (red) scale. (*D*) Detailed view of a 30-Mb region (yellow boxes in *A–C*). This genomic region and the contained genes are plotted twice in the top and the bottom lanes. In the track of Futra pairs, each blue line linking a gene from the top lane to a gene at the bottom indicates a Futra pair. In the track of iMARGI read pairs in HEK cells, each green line indicates an iMARGI read pair with the RNA end mapped to the genomic position in the top lane and the DNA end mapped to the bottom lane.

We visualized the frequency of Futra pairs in a 2D heatmap, which we call a “fusion map” (Fig. 3*C* and *SI Appendix*, Fig. S5*A*). The 2D distribution was not uniform, with more intrachromosomal than interchromosomal gene pairs (odds ratio = 27.91, *P* value <2.2 × 10^−16^, χ^2^ test). A total of 8,891 and 6,253 Futra pairs were intra- and interchromosomal, respectively. Chromosomes 1, 12, and 17 harbored the largest amounts of intrachromosomal gene pairs (*SI Appendix*, Fig. S5*B*). Chromosomes 1, 11, 12, 17, and 19 contribute to the largest amounts of interchromosomal gene pairs (*SI Appendix*, Fig. S5*C*). Higher density of gene pairs appeared on the diagonal line of the fusion map, suggesting enrichment of gene pairs within chromosomes or large chromosomal domains (Figs. 3*C* and 4*A*). We quantified the relative distances between the Futra pairs. The number of intrachromosomal Futra pairs negatively correlated with their chromosomal distance (Fig. 2*A*, purple circles). Taken together, Futra pairs exhibited nonuniform distribution in the genome, characterized by enrichment of intrachromosomal pairs and preference to smaller genomic distances.

### Differences Between the Genomic Locations of Futra Pairs and Genome Interactions.

We asked to what extent Futra pairs may correlate with genome interactions. Forty-one percent (6,253 of 15,144) of Futra pairs were interchromosomal, whereas less than 15% of chromosomal conformation capture-derived read pairs were interchromosomal (13, 14). The intrachromosomal Futra pairs exhibited enrichment in large chromosomal domains (Figs. 3*C* and 4*A* and *SI Appendix*, Fig. S6*A*). These enriched chromosomal domains ranged from approximately 1/10th to 1/3rd of a chromosome in lengths, which are ∼10–20 times the typical sizes of topologically associated domains (TADs) (15). Taken together, Futra pairs exhibited different global distribution characteristics from those of genome interactions.

### Genome-Wide Colocalization of Futra Pairs and RNA–DNA Interactions.

We asked to what extent Futra pairs may coincide with genome-wide RNA–DNA interactions. We carried out a visualized comparison of the 2D distribution of Futra pairs with that of RNA–DNA interactions (Figs. 1*E* and 3*C* and *SI Appendix*, Fig. S1*D*) and observed pronounced similarities (Fig. 4 *A*–*C* and *SI Appendix*, Fig. S6 *A–C*). For example, a set of 34 Futra pairs was enriched in an ∼7-Mb region on chromosome 9 (chr9) (32–39 Mb, Fig. 4 *A* and *D*). This Futra-pair–enriched region colocalized with a chromosomal region enriched in RNA–DNA interactions (Fig. 4 *B*–*D*). Such colocalizations were observed in multiscale analyses using different resolutions, including 10-Mb (*SI Appendix*, Fig. S6 *A–C*), 1-Mb (Fig. 4 *A*–*C*), and 100-kb (Fig. 4*D*) resolutions, as well as at the resolution of individual fusion pairs and read pairs (*SI Appendix*, Fig. S7). Four fusion transcripts, KMT2C-AUTS2, KMT2C-CALN1, KMT2C-CLIP2, and KMT2C-GTF2IRD, were formed between the KMT2C mRNA near the 152-Mb location of chromosome 7 (chr7: 152,000,000) and four mRNAs that were approximately 80 Mb away (chr7: 66,000,000–78,000,000) (Futra pairs; *SI Appendix*, Fig. S7). Correspondingly, a total of 73 RNA–DNA iMARGI read pairs were mapped to KMT2C and the four fusion partners in HEK cells (iMARGI; *SI Appendix*, Fig. S7).

We quantified the overlaps between Futra pairs and iMARGI-identified RNA–DNA interactions. Among the 6,253 interchromosomal Futra pairs, 3,014 (48.2%) overlapped with RNA–DNA interactions in either HEK or HFF cells (odds ratio = 14.1, *P* value <2.2 × 10^−16^, χ^2^ test). Among the 8,891 intrachromosomal Futra pairs, 7,427 (83.5%) overlapped with RNA–DNA interactions in either HEK or HFF cells (odds ratio = 35.44, *P* value <2.2 × 10^−16^, χ^2^ test). These data pointed to a common feature of cancer-derived Futra pairs, which is their colocalization with RNA–DNA interactions in normal cells.

### Cancer-Derived Futra Pairs That Colocalize with RNA–DNA Interaction in Normal Cells Do Not Form Fusion Transcripts in Normal Cells.

A model that may explain the colocalization of RNA–DNA interactions and Futra pairs is that RNA–DNA interactions in the normal cells poise for creation of fusion transcripts. Recognizing that this model cannot be tested by perturbation due to the very small likelihood for a fusion transcript to occur in a cancer sample, we carried out two other tests. First, we tested whether the cancer-derived Futra pairs were detectable in normal cells. We reanalyzed the merged RNA-seq datasets of more than 75 million 2 × 100-bp paired-end read pairs from HEK293T cells (16) and ran STAR-Fusion (17) on these datasets, which reported a total of 8 Futra pairs. None of the previously derived 15,144 Futra pairs from TCGA RNA-seq data were detected in HEK293T cells. In addition, we specifically tested for EML4-ALK fusion transcripts, which were reported in nonsmall cell lung carcinoma (NSCLC) (18), and there were RNA–DNA interactions between EML4 RNA and the ALK genomic locus in HEK and HFF cells (see Fig. 6*A*). Neither PCR nor quantitative PCR analysis detected EML4-ALK fusion transcripts in HEK293T cells (*SI Appendix*, Fig. S8), whereas both assays detected fusion transcripts in a NSCLC cell line (H2228) (*SI Appendix*, Fig. S8). Taken together, these data suggest that, although cancer-derived Futra pairs colocalized with RNA–DNA interactions in normal cells, the fusion transcripts found in cancer are not present in the normal cells.

### RNA–DNA Interactions in Normal Cells Are Predictive of Fusion Transcripts in New Cancer Samples.

**Fig. 5.**
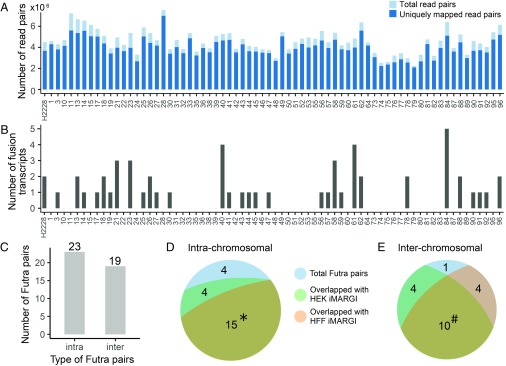
Fusion transcripts detected from the new lung cancer samples. (*A*) The number of RNA-seq read pairs (light blue bar) and the uniquely mapped read pairs (dark blue bar) of each sample (column). (*B*) Number of detected fusion transcripts (*y* axis) in each sample (columns). (*C*) Numbers of intra- and interchromosomal Futra pairs detected from the 69 cancer samples. (*D* and *E*) Intersections of these intrachromosomal (*D*) and interchromosomal (*E*) Futra pairs to RNA–DNA interactions in HEK (green) and HFF (pink) cells. *: The 15 intrachromosomal Futra pairs that overlap with RNA–DNA interactions in both HEK and HFF (yellow-green) cells are ALK:EML4, RP11-557H15.4:SGK1, FRS2:NUP107, ACTN4:ERCC2, LRCH1:RP11-29G8.3, CUX1:TRRAP, CEP70:GSK3B, NIPBL:WDR70, KCTD1:SS18, CHST11:NTN4, RP1-148H17.1:TOP1, LMO7:LRCH1, NAV3:RP1-34H18.1, LPP:PPM1L, and MTOR-AS1:RERE. #: The 10 interchromosomal Futra pairs that overlap with RNA–DNA interactions in both HEK and HFF (yellow-green) cells are LIN52:PI4KA, LPP:OSBPL6, FCGBP:MT-RNR2, KMT2B:MALAT1, ETV6:TTC3, KTN1:MALAT1, FLNA:MALAT1, COL1A2:MALAT1, COL1A1:MALAT1, and MALAT1:TNFRSF10B.

Next, we tested whether the RNA–DNA interactions in normal cells are predictive of fusion transcript formation in cancer. To this end, we analyzed a validation cohort comprising 96 new lung cancer samples from patients who were not part of the TCGA cohorts. We also analyzed a NSCLC cell line (H2228). RNA was extracted and targeted RNA-seq was carried out with Illumina’s TruSight RNA Pan-Cancer Panel. Of these 96 samples, 27 did not yield sufficient RNA for sequencing, whereas the other 69 samples produced a sequencing library and yielded on average 3.9 million uniquely aligned read pairs per sample (Fig. 5*A*). STAR-Fusion (17) was applied to this dataset and it reported a total of 42 fusion transcripts from these 69 samples (Fig. 5 *B* and *C*). These 42 fusion transcripts included EML4-ALK and FRS2-NUP107 fusions, which were also reported from the 9,966 TCGA cancer samples, as well as 40 new fusion transcripts that were not previously documented. The small amount of recurring Futra pairs between these additional cancer samples and TCGA samples is expected from the small fraction of recurring Futra pairs across the TCGA samples (Fig. 3*B*).

Among these 42 Futra pairs detected from the validation cohort, 37 (88.1%) colocalized with RNA–DNA interactions in the assayed normal cells, supporting the idea that RNA–DNA interactions in the already assayed normal cells are predictive of Futra pairs in cancer (odds ratio = 106.51, *P* value <2.2 × 10^−16^, χ^2^ test). We asked whether only intrachromosomal Futra pairs colocalized with RNA–DNA interactions. Nineteen of the 42 (45%) detected Futra pairs were interchromosomal (Fig. 5*C*), comparable to the proportion (41%) of interchromosomal Futra pairs detected from TCGA samples. Eighty-three percent (19 of 23) of intrachromosomal and 95% (18 of 19) of interchromosomal Futra pairs overlapped with RNA–DNA interactions (Fig. 5 *D* and *E*), suggesting that the colocalization of RNA–DNA interactions and Futra pairs was not restricted to intrachromosomal interactions. Taken together, the colocalization of Futra pairs and RNA–DNA interactions, the lack of cancer-derived fusion transcripts in normal cells, and the predictability of additional Futra pairs in new cancer samples support the model where RNA–DNA interactions in normal cells poise for creation of fusion transcripts in cancers. Hereafter, we refer to this model as the RNA-poise model. We call the gene pairs with RNA–DNA interactions in normal cells fusion-susceptible pairs.

### RNA–DNA Interaction Between EML4 and ALK Correlates with an RNA Fusion Without Fusion Gene in Tumor.

**Fig. 6.**
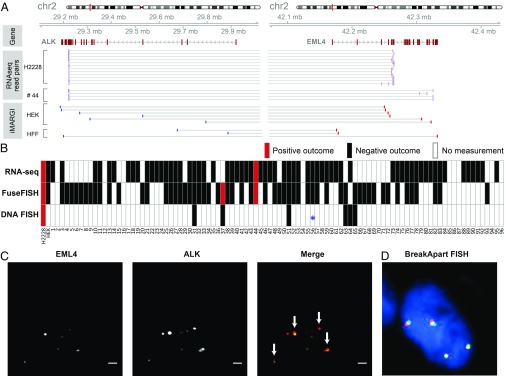
RNA fusion and DNA break. (*A*, *Middle* tracks) RNA-seq read pairs (purple bars) aligned to ALK (*Left*) and EML4 (*Right*). Each pair of paired-end reads is linked by a horizontal line. (*A*, *Lower* tracks) iMARGI read pairs aligned to the two genes. Red bars: RNA end. Blue bars: DNA end. Thin gray lines: pairing information of iMARGI read pairs. (*B*) Positive (red) and negative (black) outcomes of detection of EML4-ALK fusion transcripts based on RNA-seq, FuseFISH, and DNA FISH (ALK Break Apart FISH, a DNA rearrangement test) in each cancer sample (column). White box: no measurement. *: Detected a partial deletion of ALK gene without rearrangement. (*C*) FuseFISH images of the no. 37 cancer sample from the EML4 and ALK channels. Arrows: colocalized FISH signals indicating fusion RNA. (Scale bar: 2 μm.) (*D*) A representative image of ALK Break Apart FISH, with colocalized red and yellow signals that indicate integral ALK gene without rearrangement.

We tested whether genome rearrangement is a prerequisite step for the creation of fusion transcripts from fusion-susceptible pairs by choosing EML4-ALK fusion transcripts for this test because EML4-ALK is a fusion-susceptible pair (Fig. 6*A*), EML4-ALK fusion transcripts are detected in one of our new tumor samples (sample no. 44) (Fig. 6*B*), and there is an FDA-approved diagnosis kit (Vysis ALK Break Apart FISH) based on DNA FISH detection of the EML4-ALK fusion gene. Break Apart assays were performed by Knight Diagnostic Laboratories at the Oregon Health & Science University according to standardized protocols. We subjected the remaining tissue from sample no. 44 to DNA FISH analysis. None of our eight attempts yielded any DNA FISH signal in the remaining tissue from either control or ALK probes. We therefore could not ascertain whether there was genome rearrangement in the only sample with detectable EML4-ALK fusion transcripts.

To identify other cancer samples that express EML4-ALK fusion transcripts, we reanalyzed our collection of 96 lung cancer samples with FuseFISH, a single-molecule fluorescence in situ hybridization (sm-FISH)–based method for the detection of fusion transcripts (19, 20). We carried out quantum dot-labeled sm-FISH (21) by labeling EML4 and ALK transcripts with quantum dots at 705 nm and 605 nm, respectively (*SI Appendix*, Fig. S9). The FISH probes were designed to hybridize to the consensus exons shared among all 28 variants of EML4-ALK fusion transcripts that have been identified to date (22). Following prior literature (19, 20), fusion transcripts were detected by the colocalized sm-FISH signals targeting EML4 and ALK transcripts. In a positive control test, an average of 12 colocalized sm-FISH signals per cell were detected in a total of 22 H2228 cells (*SI Appendix*, Fig. S9) that were known to express EML4-ALK fusion transcripts (*SI Appendix*, Fig. S8) (23). In contrast, HEK293T cells exhibited on average zero colocalized signals per cell from 19 cells (*SI Appendix*, Fig. S9), consistent with the lack of such a fusion transcript in HEK293T cells (*SI Appendix*, Fig. S8).

In our collection of 96 tumor samples, only 57 had remaining tissues for FuseFISH analysis. These 57 samples included 39 that yielded RNA-seq data and 18 that did not yield RNA-seq data (Fig. 6*B*). The FuseFISH analysis detected EML4-ALK fusion transcripts in 2 samples, including sample no. 44 which was also analyzed by RNA-seq and sample no. 37 which did not yield RNA-seq data (Fig. 6 *B* and *C*). To test whether sample no. 37 had ALK-related fusion genes, we subjected it together with 6 other randomly selected samples (nos. 18, 51, 56, 57, 63, and 65) to DNA recombination analysis using Vysis ALK Break Apart FISH. None of these 7 samples exhibited ALK- related fusion genes. More specifically, 1 sample (no. 57) failed to generate DNA FISH signals from four attempts and 1 sample (no. 56, negative for EML4-ALK fusion transcripts by RNA-seq and FuseFISH analyses) exhibited a partial deletion of the ALK gene, but no sign of ALK-related fusion genes (Fig. 6*B*, *). The other 5 samples, including sample no. 37, exhibited integral ALK genes (Fig. 6 *B* and *D*). Taken together, the lung cancer sample no. 37 expressed EML4-ALK fusion transcripts without having an EML4-ALK fusion gene. These data suggest that genome rearrangement is not a prerequisite step for the creation of fusion transcripts from fusion-susceptible pairs. In other words, the RNA-poise model does not require alterations of the DNA.

## Discussion

### Abundance of Genome Rearrangement-Independent Fusion Transcripts.

Our RNA FISH and DNA FISH analyses revealed a cancer sample that contained a fusion transcript without the corresponding fusion gene. Such an example, although not often seen in the literature, may not be a rare case (11). The lack of reports is likely attributable to the research attention paid to the other side of the coin, i.e., the fusion transcripts created by fusion genes (2). Indeed, ∼36–65% of fusion transcripts derived from cancer RNA-seq data were attributable to genome rearrangement (Low Pass bars, figure S1A of ref. 1). However, this is likely an overestimate because when low-quality whole-genome sequencing (WGS) data were removed, only ∼30–45% of fusion transcripts had corresponding WGS reads (High Pass bars, figure S1A of ref. 1). These published results are consistent with the notion that fusion genes do not account for all observed fusion transcripts and suggest the occurrence of fusion transcripts independent of genome rearrangement. However, we recognize that to date, the sheer amount of validated fusion RNAs independent of genome rearrangement remains limited, which warrants future investigation.

### The RNA-Poise Model Allows for Splicing Errors.

Fusion transcripts can be created by two processes. The better-recognized process is through transcription of a fusion gene that was created by genome rearrangement. The less-recognized process is by RNA splicing errors, where two separate transcripts were spliced together (transsplicing) (24). Transsplicing does not involve genome rearrangement. A theoretical gap in the splicing error model is that transsplicing can happen only to two RNA molecules that are close to each other in 3D space; however, except for neighboring genes (11), the chances for two RNA molecules transcribed from distant chromosomal locations to meet in space are small. Therefore, it remains difficult to perceive a biophysical process in which fusion transcripts are created by splicing errors.

The RNA-poise model fills this theoretical gap. The preinstallation of gene 1’s transcripts on gene 2’s genomic sequence positions gene 2’s nascent transcripts spatially close to gene 1’s transcripts, allowing for the possibility of transsplicing. Furthermore, the majority of splicing events are cotranscriptional. The availability of transcripts of gene 1 during gene 2’s transcription allows for the opportunity to perform cotranscriptional transsplicing.

### Breaking Down the RNA-Poise Model by RNA–DNA Interactions.

**Fig. 7.**
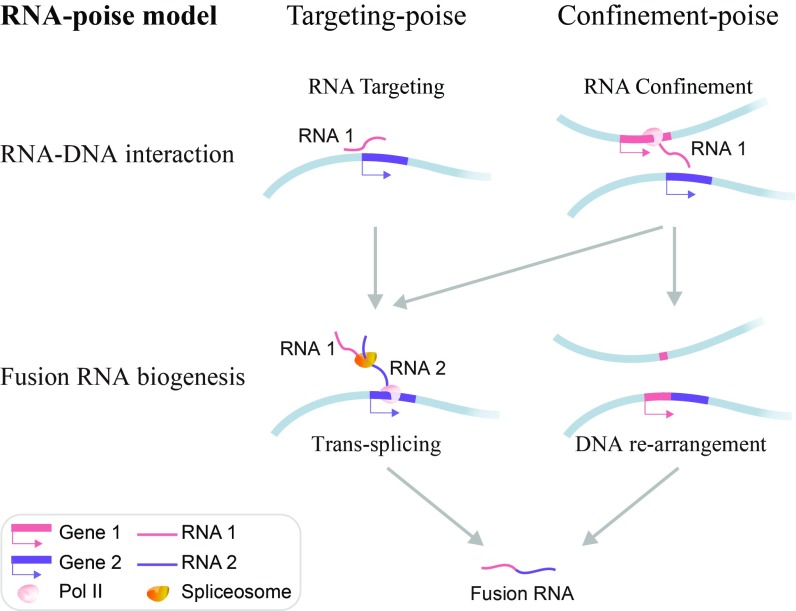
RNA-poise model. In this model, the transcripts of one gene (RNA 1, red bar) can exhibit spatial proximity to another gene (RNA 2, purple bar) due to tethering (RNA targeting) or spatial proximity of the two genes (RNA confinement). Both cases could enhance splicing errors (gray arrows), whereas the proximity of genomic sequences may also facilitate gene fusion (gray arrow on the right), which subsequently produces fusion RNA.

Remote RNA–DNA interactions could be created by at least two means. First, the caRNA can target specific genomic sequences, which could be mediated by tethering molecules (RNA targeting, Fig. 7). Second, the spatial proximity of the genomic sequences in 3D space could bring the nascent transcripts of one gene to the genomic sequence of another gene (RNA confinement, Fig. 7). Both means of RNA–DNA interactions provide spatial proximity between two RNA molecules and thus allow for splicing errors. In addition, the spatial proximity of two genes in the RNA confinement model could enhance the chances of genome rearrangement of the spatially close genomic sequences and thus create fusion genes (25). Thus, the RNA-poise model can be regarded as a union of two submodels, depending on the process of RNA–DNA interaction. One submodel (targeting-poise model, Fig. 7) could create fusion transcripts only by transsplicing. The other submodel (confinement-poise model, Fig. 7) could create fusion transcripts by either transsplicing or creation of fusion genes.

## Materials and Methods

### Reference Genome and Gene Annotations.

Human genome assembly hg38/GRCh38 and Ensembl gene annotation release 84 (GRCh38.84) were used throughout all data analyses.

### Public RNA-Seq Data.

HEK293T RNA-seq datasets were downloaded from the NCBI BioProject database (accession nos. SRR2992206–SRR2992208 under project no. PRJNA305831) (16). The three datasets were merged.

### TCGA Derived Fusion Transcripts.

The TCGA RNA-seq–derived fusion transcripts were downloaded from the Tumor Fusion Gene Data Portal (www.tumorfusions.org) (26). Tier 1 and tier 2 fusion transcripts were used in our analyses. Genomic coordinates were converted to hg38 by liftOver. Following Davidson et al. (8), Futra pairs within 200 kb on hg38 were removed. The data of Futra pairs were mainly processed using R (27) with Bioconductor packages GenomicRanges (28) and InteractionSet (29).

### Visualization of Futra Pairs.

Heatmaps of the count matrix were plotted using Bioconductor package ComplexHeatmap (30). Genomic plots of Futra pairs were created with GIVE (31).

### Constructing iMARGI Sequencing Libraries.

#### Nuclei preparation and chromatin digestion.

Approximately 5 × 10^6^ cells were used for the construction of an iMARGI sequencing library. Cells were cross-linked in 1% formaldehyde at room temperature (RT) for 10 min with rotation. The cross-linking reaction was quenched with glycine at 0.2 M concentration and incubated at RT for 10 min. Cells were pelleted, washed using 1× PBS, and aliquoted into ∼5 × 10^6^ in each tube. To prepare nuclei, cross-linked cells were incubated in 1 mL of cell lysis buffer (10 mM Tris⋅HCl, pH 7.5, 10 mM NaCl, 0.2% Nonidet P-40, 1× protease inhibitor) on ice for 15 min and homogenized with dounce homogenizer pestle A for 20 strokes on ice. Nuclei were pelleted and weighed to estimate the pellet volume (10 mg of nuclei pellet was estimated to be about 10 μL). The nuclei pellet was incubated with SDS buffer (0.5× Cutsmart buffer, 0.5% SDS) at 1:3 vol ratio and 62 °C for 10 min with mixing and immediate quenching with a final 1% of Triton X-100. To digest chromatin, the washed nuclei pellet was resuspended in an AluI chromatin digestion mix [2.3 units/μL AluI (NEB), with 0.3 unit/μL RNasinPlus (Promega) and 1× Cutsmart buffer] and incubated at 37 °C overnight with mixing. After chromatin digestion, 1 μL of RNase I (1:10 diluted in 1× PBS) was directly added to the reaction mixture and incubated at 37 °C for 3 min to fragment RNA. Nuclei were pelleted and washed twice using PNK wash buffer (20 mM Tris⋅HCl, pH 7.5, 10 mM MgCl_2_).

#### Ligations.

To prepare the RNA and DNA ends for linker ligation, nuclei were incubated in 200 μL RNA 3′-end dephosphorylation reaction mix [0.5 unit/μL T4 PNK (NEB), 0.4 unit/μL RNasinPlus, 1× PNK phosphatase buffer, pH 6.5] at 37 °C for 30 min with mixing. Nuclei were washed twice with PNK buffer, resuspended in 200 μL DNA A-tailing mix [0.3 unit/μL Klenow Fragment (3′→5′ exo-) (NEB), 0.1 mM dATP, 0.1% Triton X-100, 1× NEB buffer 2] and incubated at 37 °C for 30 min with mixing. The same linker sequence as described in the previous MARGI paper was used (10). For in situ RNA-linker ligation, nuclei were resuspended in 200 μL ligation mix [38 μL adenylated and annealed linker, 10 units/μL T4 RNA ligase 2-truncated KQ (NEB), 1× T4 RNA ligase reaction buffer, 20% PEG 8000, 0.1% Triton X-100, 0.4 unit/μL RNasinPlus] and incubated at 22 °C for 6 h and then 16 °C overnight with mixing. After ligation, the nuclei were washed five times with PNK buffer to remove excess free linker. For in situ RNA–DNA proximity ligation, nuclei were resuspended in 2 mL of proximity ligation mixture [4 units/μL T4 DNA ligase (NEB), 1× DNA ligase reaction buffer, 0.1% Triton X-100, 1 mg/mL BSA (NEB), 0.5 unit/μL RNasinPlus] and incubated at 16 °C overnight.

#### Library construction.

To reverse cross-linking, nuclei were washed twice with 1× PBS, resuspended in 250 μL of extraction buffer [1 mg/mL Proteinase K (NEB), 50 mM Tris⋅HCl, pH 7.5, 1% SDS, 1 mM EDTA, 100 mM NaCl] and incubated at 65 °C for 3 h. DNA and RNA were extracted by adding an equal volume of phenol:chloroform:isoamyl alcohol (pH 7.9, Ambion) followed by ethanol precipitation. The subsequent steps including removal of biotin from nonproximity ligated linkers, pulldown of RNA–DNA chimera, reverse transcription of RNA, DNA denaturation, circularization, oligo annealing and BamHI (NEB) digestion, and sequencing library generation were performed as previously described (10). iMARGI libraries were subsequently subjected to paired-end 100-cycle sequencing on an Illumina HiSeq 4000. The circularization and library construction strategy can phase RNA and DNA ends into Read 1 and Read 2 as shown Fig. 1*A*, which is the same as with MARGI library configuration (10). Since AluI restriction enzyme recognizes “AGCT” sequence and leaves “CT” at the 5′ end of the cut, we expect the first two bases of DNA end (Read 2) to be enriched with CT.

### Analysis of iMARGI Sequencing Data.

#### Mapping iMARGI read pairs.

The detailed iMARGI data-processing methods can be found in our GitHub repository (https://github.com/Zhong-Lab-UCSD/iMARGI_methods). Briefly, they include three main steps. First, the read pairs were cleaned by in-house scripts. According to the library construction design, read pairs were filtered out if the 5′-most two bases of their DNA end (Read 2) were not CT. In addition, the first two bases of the RNA end (Read 1) were removed as they are random nucleotides. Then, the cleaned read pairs were mapped to the human genome (hg38), using bwa mem (version 0.7.17) with parameters “-SP5M” (32). Finally, pairtools (version v0.2.0, https://github.com/mirnylab/pairtools) and in-house scripts were used to parse, deduplicate, and filter the mapped read pairs. The valid read pairs that were mapped to genomic locations within 200 kb of each other were defined as proximal interactions, which were excluded from our analysis. GenomicRanges (28) and InteractionSet (29) were used for further analysis of iMARGI data.

#### Visualization of iMARGI read pairs.

Heatmaps of the count matrix were plotted using Bioconductor package ComplexHeatmap (30). Genomic plots of iMARGI read pairs were created with Bioconductor package Gviz (33) and GIVE (31).

#### Intersection of iMARGI read pairs and Futra pairs.

An iMARGI read pair was regarded as overlapping with a Futra pair when the RNA end was strand-specifically mapped to the gene body of one gene in the Futra pair and the DNA end was mapped to the gene body ±100 kb flanking regions of the other gene in the Futra pair.

### FuseFISH Analysis.

#### Probe design.

Oligonucleotide probes were designed to hybridize to exons 2–6 of EML4 RNA and exons 20–23 of ALK RNA. These exons were chosen because they were present in all of the observed variations of EML4-ALK fusion genes. These probes were 35–40 nt in length, with similar GC contents and melting temperatures.

#### Conjugation of quantum dots to oligonucleotide probes.

Oligos were modified on the 5′ end with a primary amino group and a spacer of 30 carbons to minimize steric hindrance of probe–RNA hybridization. These probes were conjugated with quantum dots through the amino group using EDC reaction (34). The probes were subsequently purified with 0.2 μm membrane filtration and 100,000 molecular weight cutoff (MWCO). The retentate of the 100,000 MWCO was subjected to dynabeads MyOne SILANE purification to remove any remaining unconjugated probes. A subsequent 0.2-μm membrane filtration was used to remove any final aggregates.

#### Hybridization of adherent cell lines.

Probe hybridization in H2228 cells was carried out as previously described (19, 21). Briefly, probes were added to the hybridization solution and incubated with the cells at 37 °C overnight. Cells were washed and resuspended in 1× PBS for imaging.

#### Hybridization of tissue samples.

Probe hybridization in tissues was carried out as previously described (35). Briefly, a hybridization solution with probes was added to the surface of the parafilm to form droplets. A tissue slice (5–10 μm in thickness) fixed on a glass coverslip was gently placed over the hybridization solution. The mixture was incubated at 37 °C overnight. The tissue was subsequently washed with wash buffer and resuspended in 1× PBS for imaging.

#### Imaging and analysis.

Cells or tissues were imaged in 1× PBS through wide-field fluorescence imaging using an Olympus IX83 inverted microscope at 60× oil immersion objective (N.A. = 1.4). Image processing was carried out as previously described (36). Briefly, single transcripts were detected using an automated thresholding algorithm that searches for robust thresholds, where counts do not change within a range. Fusion transcripts were determined by searching for colocalization of detection transcripts by overlap between predicted centers within a radius.

### RNA Sequencing and Analysis.

RNA was extracted with Trizol from an H2228 cell line and lung cancer tissue samples of the approximate size 3 mm×3 mm× 30 μm per sample. RNA sequencing was carried out using the TruSight RNA Pan-Cancer Panel (Illumina) following the manufacterer’s protocol. All of the RNA-seq data, including HEK293T public data, the H2228 cell line, and lung cancer sample sequencing data, were mapped to the human genome (hg38) using STAR (v2.5.4b) with default parameters (37). Fusion transcripts were called using STAR-Fusion (v0.8.0) (17), requiring both numbers of supporting discordant read pair and junction-spanning read larger than zero and the sum of them larger than 2.

## Supplementary Material

Supplementary File
