# Supplementary material for: Genome-wide colocalization of RNA–DNA interactions and fusion RNA pairs
Source: Proc Natl Acad Sci U S A. 2019 Feb 4;116(8):3328–37. doi: 10.1073/pnas.1819788116 (PMC6386723; doi:10.1073/pnas.1819788116)
Supplement: Supplementary File [file pnas.1819788116.sapp.pdf]

PNAS
www.pnas.org

Supplementary Information for

Genome-wide co-localization of RNA-DNA interactions and fusion RNA pairs

Zhangming Yan, Norman Huang, Weixin Wu, Weizhong Chen, Yiqun Jiang, Jingyao Chen, Xuerui Huang, Xingzhao Wen, Jie Xu, Qiushi Jin, Kang Zhang, Zhen Chen, Shu Chien, and Sheng Zhong

Shu Chien and Sheng Zhong
E-mail: shuchien@ucsd.edu and szhong@ucsd.edu

This PDF file includes:

Figs. S1 to S9
Table S1

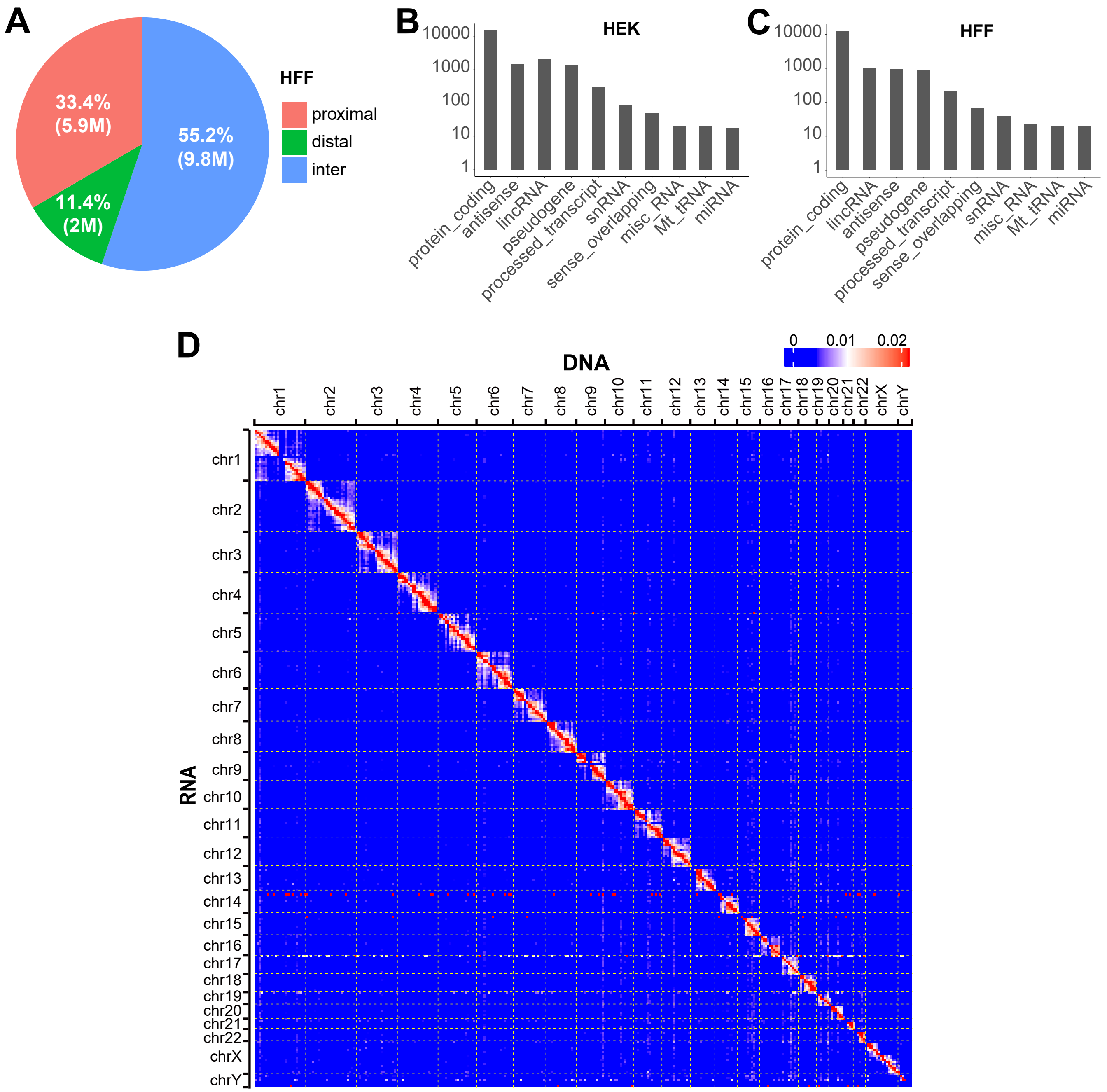

Fig. S1. Overview of iMARGI data. (A) shows proportions of proximal, distal, and inter-chromosomal read pairs in collections of valid RNA-DNA interaction read pairs in HFF cells. M: million read pairs. (B) and (C) show number of RNAs involved in remote RNA-DNA interactions with 10 or more iMARGI read pairs categorized by RNA type in HEK and HFF cells, respectively. (D) Heatmap of an RNA-DNA interaction matrix in HFF cells. The numbers of iMARGI read pairs are plotted with respect to the mapped positions of the RNA-end (row) and the DNA-end (column) from small (blue) to large (red) scale, normalized in each row.

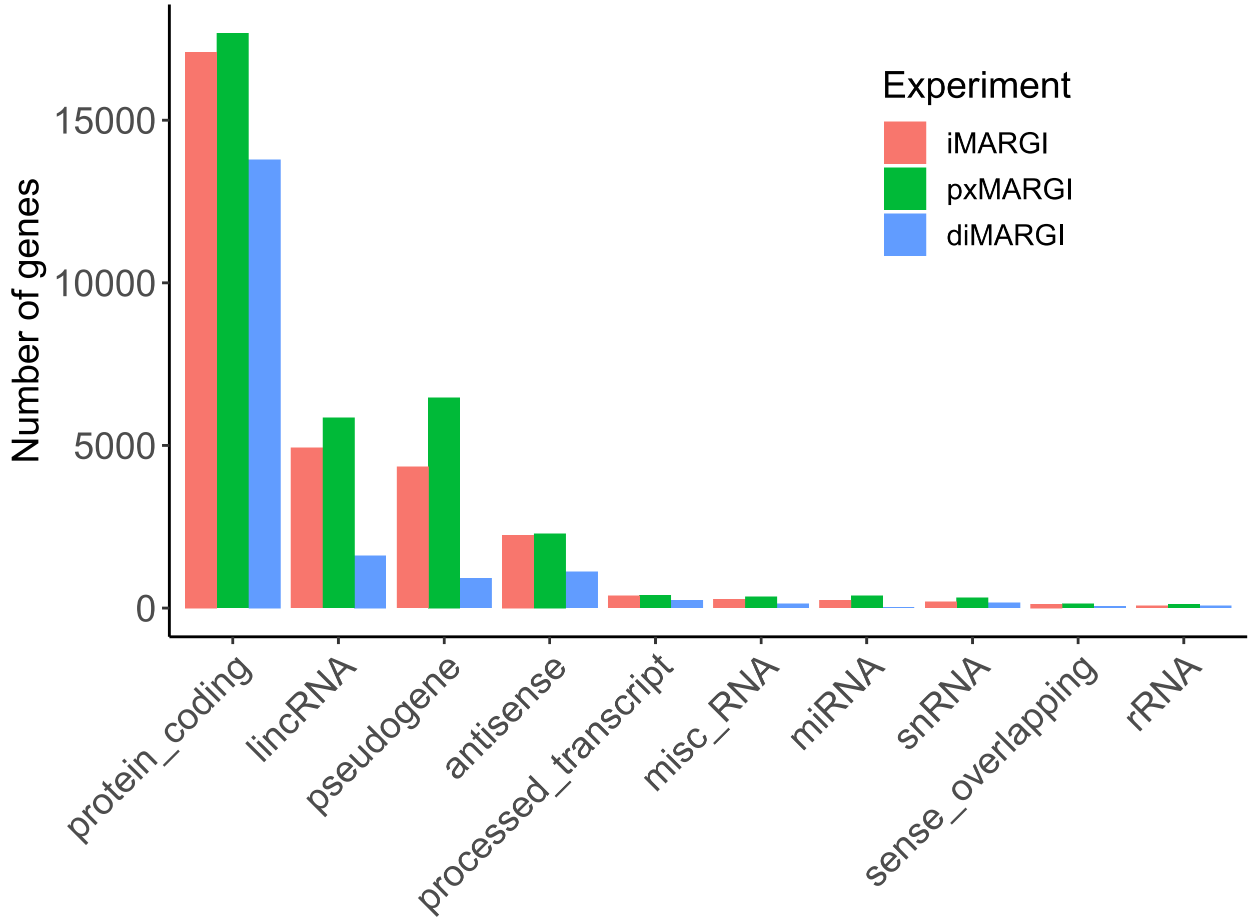

Fig. S2. Comparison of caRNAs revealed by iMARGI, pxMARGI and diMARGI. Numbers of caRNA genes (y axis) in each RNA category (x axis) are provided.

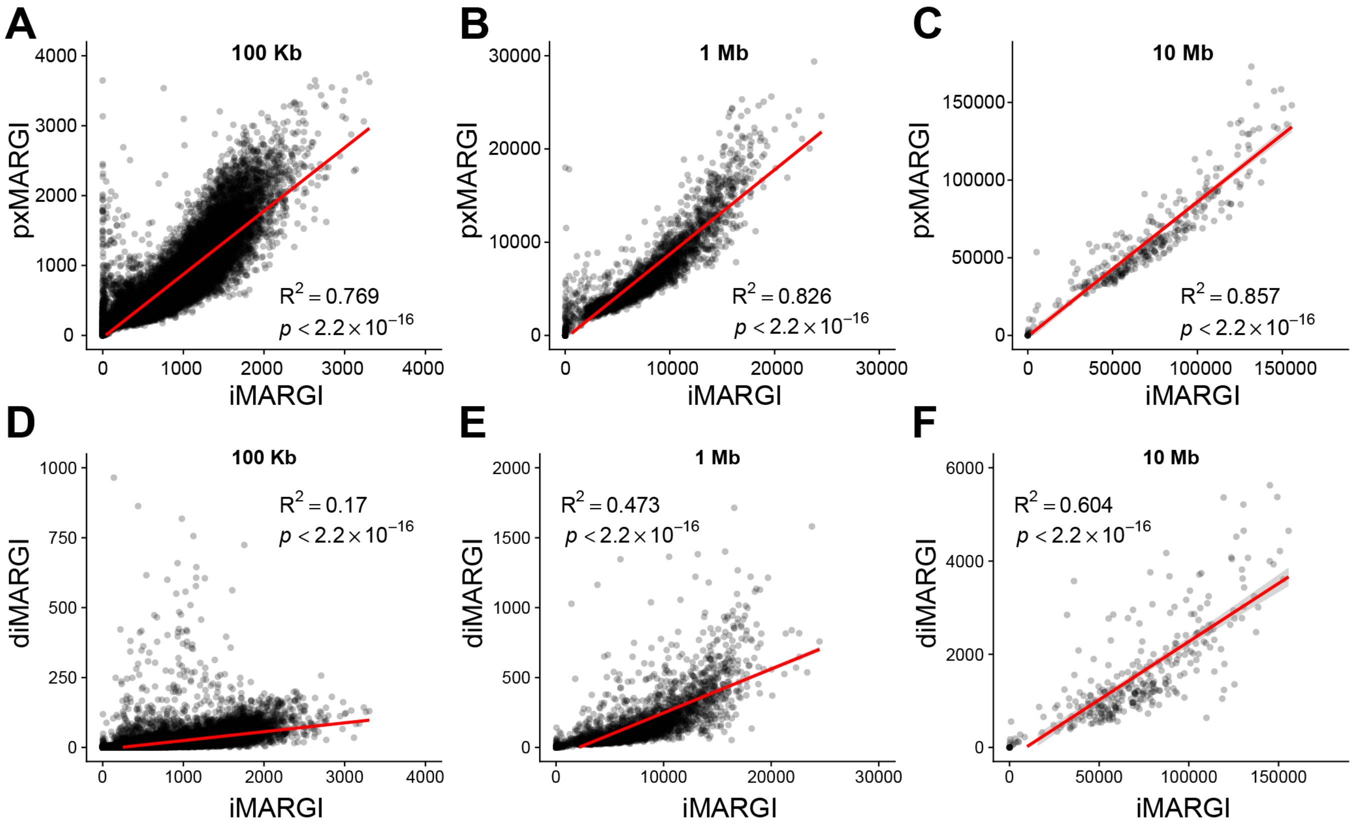

Fig. S3. Scatterplots of RNA attachment levels in every genomic segment. RNA attachment level in every bin (dot) from iMARGI (x axis) is plotted against pxMARGI (y axis, A-C) or against diMARGI (y axis, D-F). Bin sizes are 100 kb (A, D), 1 Mb (B, E), and 10 Mb (C, F).

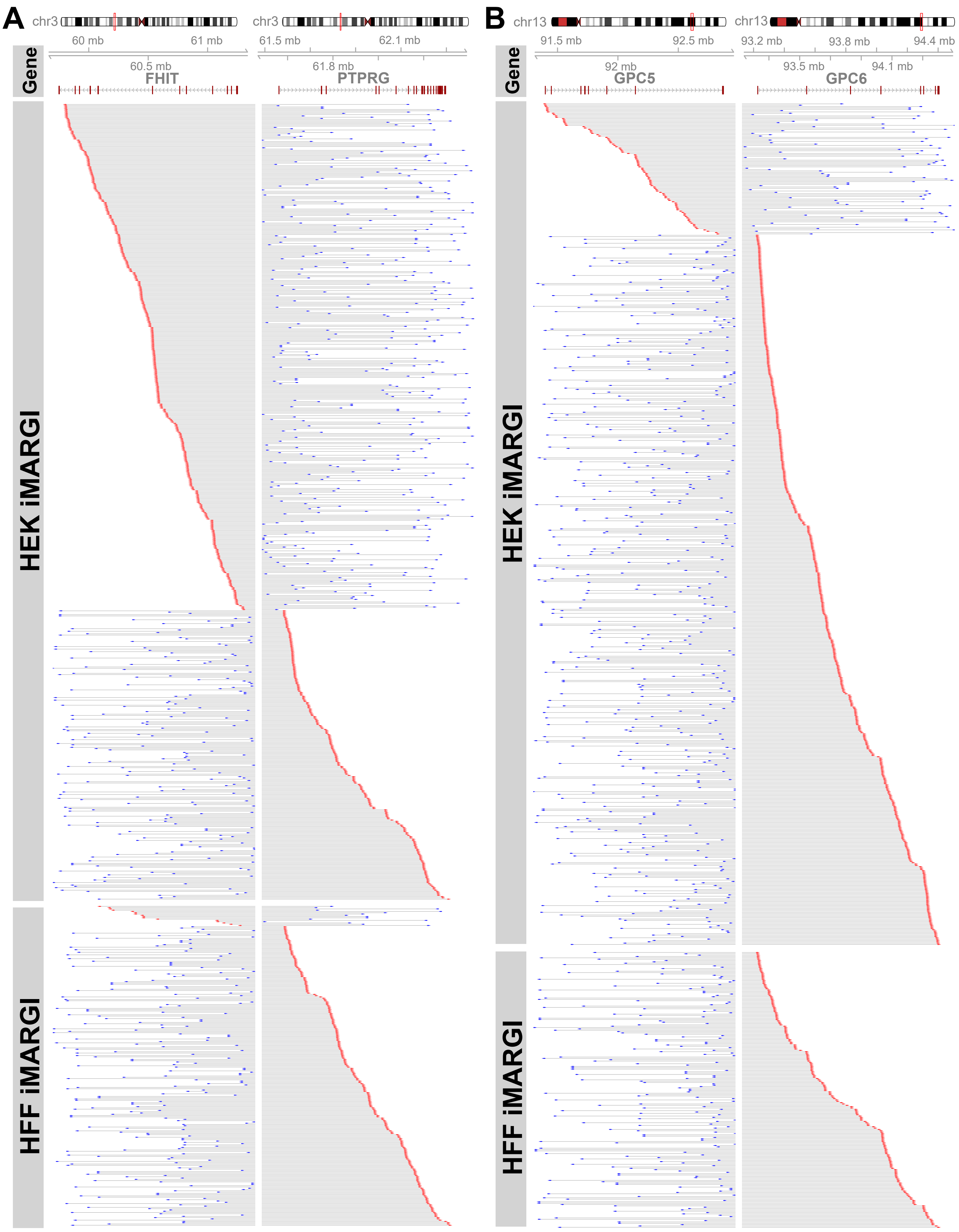

Fig. S4. Gene pairs with the largest numbers of uniquely mapped iMARGI read pairs. (A) FHIT (left) and PTPRG (right), separated by approximately 1.5 Mb in the genome. (B) GPC5 (left) and GPC6 (right), separated by approximately 2 Mb in the genome. Each read pair occupies one horizontal line. Red bars: the mapped RNA-ends. Blue bars: the mapped DNA-ends.

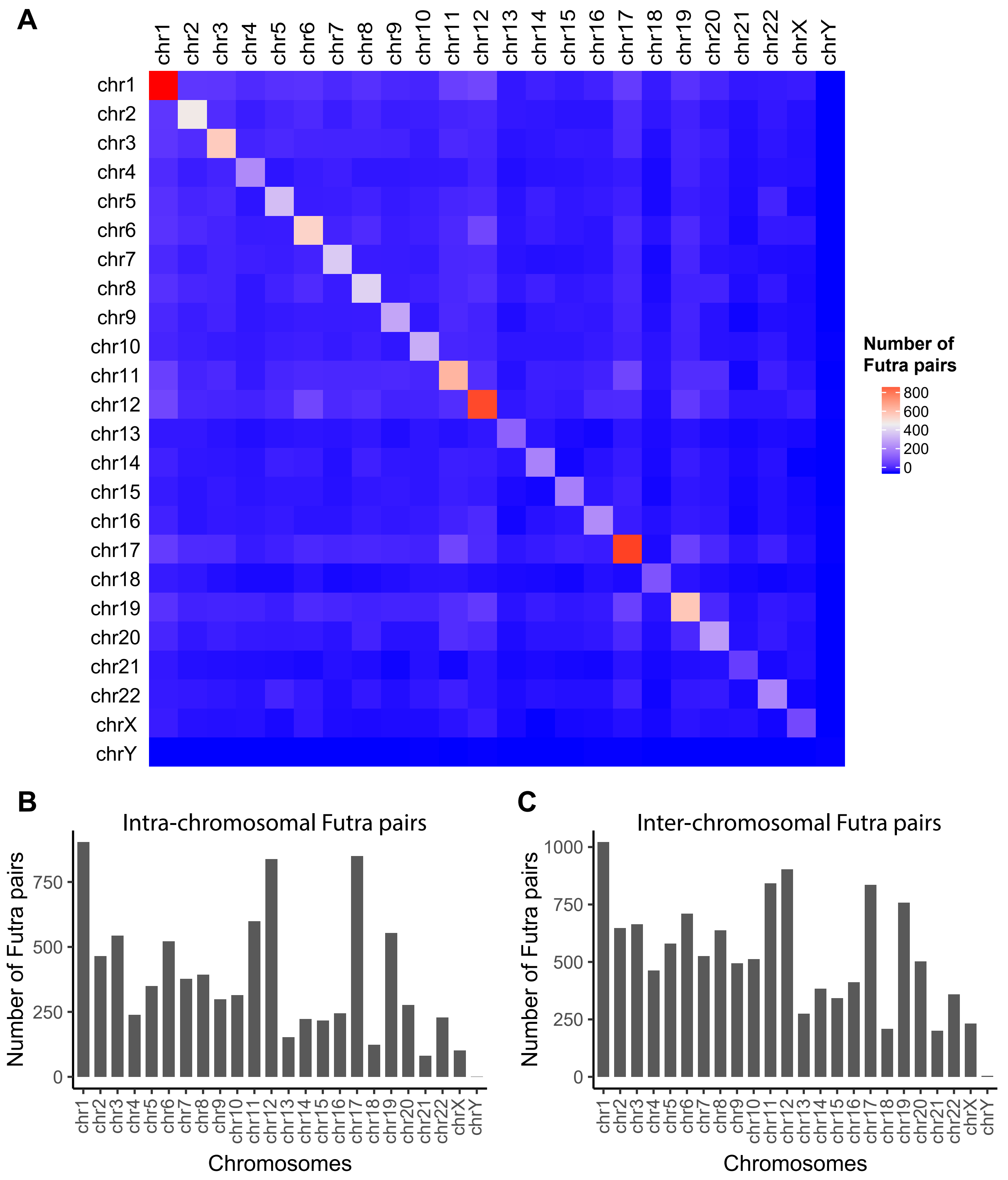

Fig. S5. Chromosomal view of the 15,144 Futra pairs derived from 33 cancer types. (A) The genomic distribution of Futra pairs. The numbers of Futra pairs of corresponding chromosome pairs are shown in a blue (small) to red (large) color scheme. (B) Chromosome by chromosome (columns) counts of intra-chromosomal Futra pairs. (C) Chromosome by chromosome (columns) counts of Futra pairs with one participating RNA mapped to this chromosome.

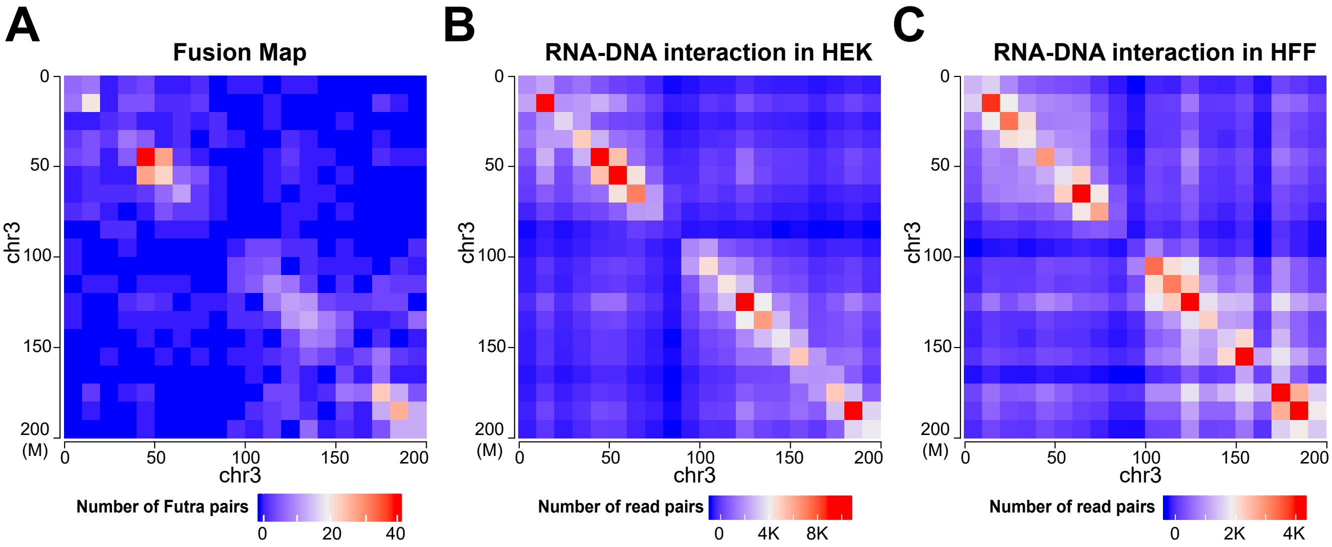

Fig. S6. Heatmaps of intra-chromosomal fusion gene pairs (A) and iMARGI read pairs of HEK (B) and HFF cells (C) in chromosome 3 at 10 Mb resolution.

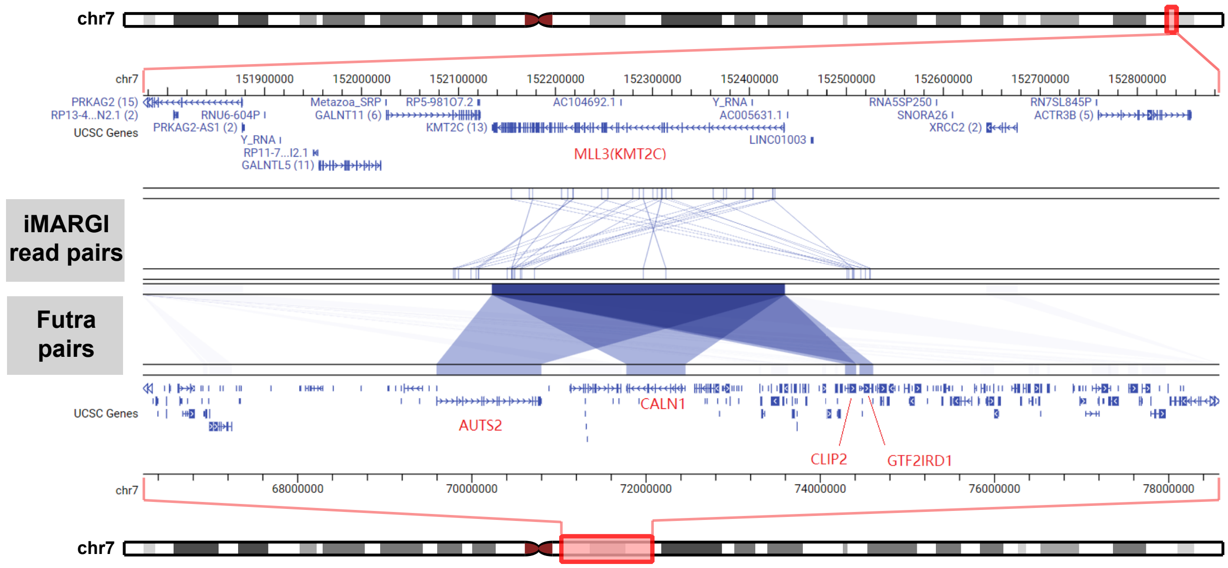

Fig. S7. Genomic view of iMARGI read pairs and Futra pairs. Top lanes correspond to the genomic region of chr7:151.8-152.9 Mb that encompass the Kmt2c (Mll3) gene. Bottom lanes correspond to the genomic region of chr7:66-78.4 Mb that include genes Auts2, Caln1, Clip2, and Gtf2ird1. Each iMARGI read pair is presented as a line in the iMARGI panel, linking mapped position of the RNA-end (top lane) and the position of the DNA-end (bottom lane). Each Futra pair is presented as a shade in the Futra panel, linking one RNA (top lane) with the other (bottom lane). The visualization was made with GIVE (www.givengine.org).

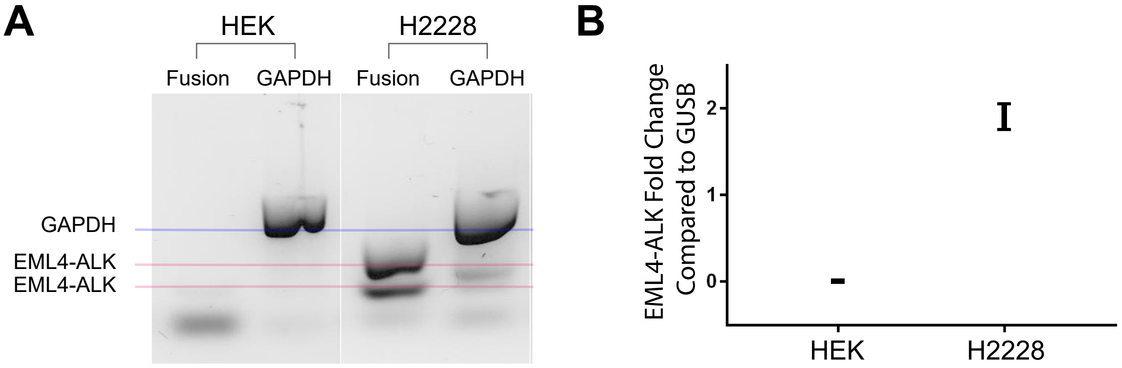

Fig. S8. Detection of fusion transcripts. (A) Northern blot analysis of GAPDH (control) and EML4-ALK fusion transcripts in HEK and a NSCLC cell line (H2228). A forward primer GCATAAAGATGTCATCATCAACCAAG against EML4 and a reverse primer TCTTGCCAGCAAAGCAGTAGTTGG against ALK were used in the Fusion lanes. A forward primer ACCACAGTCCATGCCATCAC and a reverse primer TCCACCACCCTGTTGCTGTA against GAPDH were used in the GAPDH Lanes. The two bands in the H2228 Fusion lane correspond to two known variants of the EML4-ALK fusion transcripts. (B) qPCR analysis of EML4-ALK fusion transcripts using house-keeping GUSB mRNA as the internal control. Fold change of EML4-ALK vs. GUSB (y axis) is plotted in HEK and H2228. Error bars: standard deviation derived from 4 replicates.

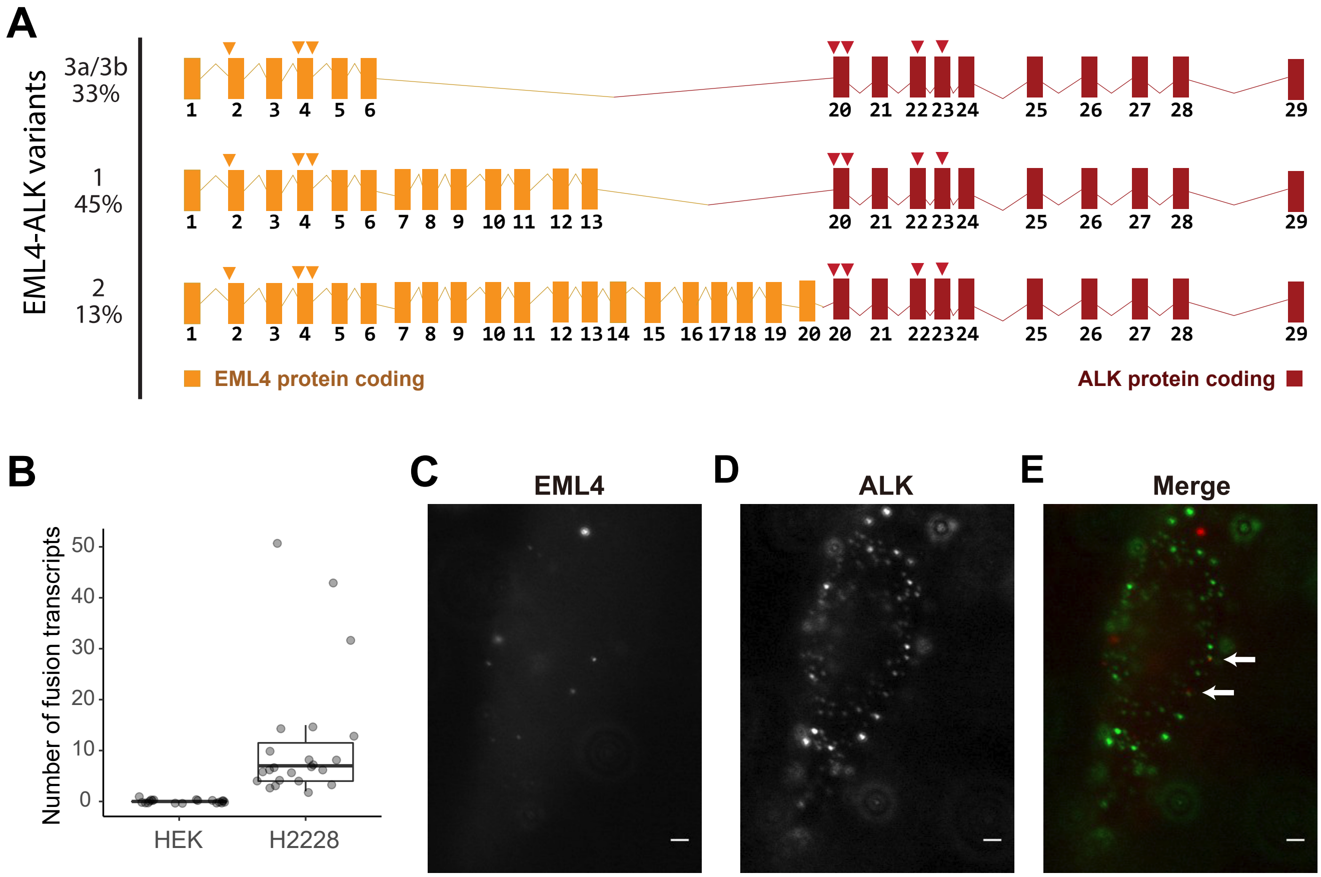

Fig. S9. FuseFISH analysis in HEK and H2228 cells. (A) Schematic view of the top three most abundant variants of EML4-ALK fusion transcripts. Each row shows a variant with its included EML4 exons (yellow boxes) and ALK exons (red boxes). Arrows: targeting locations of FISH probes. Probes labeled with Qdots of 605 nm (yellow arrows) and 705 nm (red arrows) were complementary to EML4 and ALK RNA sequences, respectively. (B) Numbers of co-localized signals (y axis) in 19 single cells (dots) of the HEK and 22 single cells (dots) of H2228. (C-E) Representative images of the EML4 (C) and ALK (D) channels and their merged image (E). Arrows: co-localized signals. Scale bar: 2 μm.

Table S1. Numbers of read pairs generated by iMARGI, pxMARGI, and diMARGI in HEK209T cells. The total number of read pairs (Total), uniquely mapped inter-chromosomal (Inter-chromosomal), and uniquely mapped distal (mapped to the same chromosomal and were separated by 20,000 bp) (Distal) read pairs were provided the columns. M: million.

	Total	Inter-chromosomal	Distal
**iMARGI**	361.2 M	18,896,538	3,787,394
**pxMARGI**	206.8 M	18,851,066	1,394,410
**diMARGi**	382.9 M	521,745	26,230
